# Expression profiles of E/P receptors and fibrosis in GnRHa-treated and -untreated women with different uterine leiomyomas

**DOI:** 10.1371/journal.pone.0242246

**Published:** 2020-11-13

**Authors:** Khaleque N. Khan, Akira Fujishita, Akemi Koshiba, Kanae Ogawa, Taisuke Mori, Hiroshi Ogi, Kyoko Itoh, Satoshi Teramukai, Jo Kitawaki

**Affiliations:** 1 Department of Obstetrics and Gynecology, Graduate School of Medical Science, Kyoto Prefectural University of Medicine, Kyoto, Japan; 2 Department of Gynecology, Saiseikai Nagasaki Hospital, Nagasaki, Japan; 3 Department of Pathology and Applied Neurobiology, Kyoto Prefectural University of Medicine, Kyoto, Japan; 4 Department of Biostatistics, Graduate School of Medical Science, Kyoto Prefectural University of Medicine, Kyoto, Japan; University of Insubria, ITALY

## Abstract

Differential expressions of estrogen/progesterone receptors (ER/PR) and individual component of extracellular matrices derived from fibroid are reported. Information on the pattern of change in ER/PR expression and amount of tissue fibrosis after hormonal treatment is unclear. We investigated pattern of change in ER/PR expression and percentage of tissue fibrosis in different uterine leiomyomas after gonadotropin-releasing hormone agonist (GnRHa) treatment. Biopsy specimens from fibroids and adjacent myometria were collected after surgery from women with submucosal myoma (SMM, n = 18), intramural myoma (IMM, n = 16) and subserosal myoma (SSM, n = 17). A proportion of women in each group of fibroid underwent treatment with GnRHa for a variable period of 3–6 months. Tissue expression of ER and PR was analyzed by immunohistochemistry. In vitro cell proliferation effect of GnRHa on human umbilical vein endothelial cells (HUVECs) was examined. Distribution of tissue fibrosis was examined by Masson’s trichrome staining with computer-captured image analysis of fibrosis derived from different types of fibroid. PR content was significantly higher than ER in tissues derived from GnRHa-untreated women with SMM and SSM (p = 0.04 for both). Comparing to untreated group, GnRHa-treatment significantly decreased either ER or PR expression in different fibroids. Exogenous treatment with GnRHa dose-dependently decreased proliferation of HUVECs. No significant difference was observed in the percentage of fibrosis in tissues collected from GnRHa-treated and -untreated women with fibroids. The distribution of fibrosis in myoma/myometria and occurrence of fibrosis in perivascular area showed an increasing trend with higher age of the women and with larger size of fibroids. Our findings suggest that despite estrogen dependency, higher PR content in GnRHa-untreated group may indicate a potential role of progesterone in leiomyoma growth. Although GnRHa therapy may shrink fibroids and reduce risk of bleeding during surgery, the occurrence of diffuse tissue fibrosis may impair effective reduction of fibroid size after hormonal treatment.

## Introduction

Uterine leiomyomyomas (also called fibroids or myomas) are the most common benign tumors of female reproductive tract. They are highly prevalent with 70–80% of women burdened by the end of their reproductive years [1]. It is difficult to establish the real incidence of fibroids because most women will not develop symptoms, thus the global impact in women’s health is underestimated [2–4]. African-American women are reported to higher leiomyoma incidence with more severe clinical symptoms compared to Caucasians [5,6]. Fibroids are a leading cause of pelvic pain, abnormal vaginal bleeding, miscarriage, and infertility [7]. It is not usually fatal but can produce serious clinical symptoms such as excessive uterine bleeding. They have been claimed as the leading indication for hysterectomy. The precise molecular and cellular changes that led to the development and growth of uterine leiomyoma are not well understood. Dysregulation of inflammatory processes are thought to be involved in the initiation of leiomyoma and extracellular matrix deposition. Tissue inflammation, cell proliferation and angiogenesis are the key cellular events implicated in leiomyoma growth [8,9].

Fibroids are hormone-depended tumors related to an environment of estrogen and progesterone, therefore, in many cases symptoms are relieved at the time of menopause [10]. Different risk factors have been established to be involved in the development of fibroids ranging from genetic predisposition to variable lifestyle behaviors [11]. In order to evaluate the response of ovarian steroids in fibroid tissue and adjacent myometrium, menstrual phase-dependent and-independent expression of estrogen receptor (ER) and progesterone receptor (PR) have been reported [12–14]. Marugo et al. [15] carefully evaluated cytosolic and nuclear ER and PR expression in uterine leiomyomas, endo-myometrium and myometrium in two types of fibroids. They found a decrease value of ER during the post-ovulatory phase whereas ER and PR were significantly more numerous in submucous than in subserosal myomas, both in the proliferative and in the secretory phase of the menstrual cycle. In this study, intramural myoma was not included. According to the report by Mozzachio et al [16], while ERα expression was variable between the myometrial and tumor tissues, PR expression was intense and diffuse throughout all tissues, with correlation between the myometrial and tumor tissues. However, detail information on the expression patterns of ER and PR in tumor and myometrial tissues derived from three different types of fibroids after hormonal treatment is scanty.

Extracellular matrix (ECM) accumulation and remodeling are thought to be crucial for fibrotic disease such as uterine leiomyoma. Fibroids are characterized by elevated levels of collagens, fibronectin, laminins, and proteoglycans [17–19]. ECM plays important role in forming bulk-structure of fibroids and ECM-rich rigid structure within these tumors that may be the cause of abnormal bleeding, pelvic pain and pressure effect. Accumulation of ECM is affected by growth factors, cytokines, steroid hormones, and microRNAs [20–25]. Among these, TGF-β and activin-A have been suggested as key players in the accumulation of excessive ECM leading to fibrosis in leiomyoma via a cascade of epithelial-mesenchymal transition (EMT), fibroblast-myofibroblast transdifferentiation (FMT), smooth muscle metaplasia (SMM) and fibrogenesis [20,22,26–28]. Some reports indicated elevated levels of ECM components and others reported distribution of fibrosis in some specific type of fibroid. Information on the parallel distribution of fibrosis in the tumor tissue and adjacent myometria in different types of fibroids such as submucosal myoma (SMM), intramural myoma (IMM), and subserosal myoma (SSM) is still lacking.

At present, several classes of compounds, such as gonadotropin-releasing hormone agonist (GnRHa, leuprolide acetate), GnRH antagonist (cetrorelix acetate), selective progesterone receptor modulators (ulipristal acetate/asoprisnil), antiprogestin (mifepristone), and natural compounds such as vitamin D and resveratrol (polyphenol extract) have been studied as medical treatments that target ECM in uterine leiomyoma [9]. Different harmful stimuli including inflammation-induced generation of reactive oxygen species (ROS) with consequent tissue stress reaction may create a chronic inflammatory state in the uterus culminating in fibrosis [29,30]. As a marker of inflammation, we reported that pattern of tissue infiltration of macrophages and prostaglandin (PG) production differs for different uterine leiomyomas [31]. We previously reported GnRH receptors expression in endometriosis, adenomyosis and uterine leiomyoma and found that GnRHa treatment was able to significantly decrease tissue inflammation, cell proliferation, angiogenesis, and tissue stress reaction in different reproductive diseases including uterine leiomyomas [32–34]. Immunohistochemical studies showed a significant decrease in the cellular proliferation index, ER and PR expression in the leuprolide acetate-treated fibroids compared with non-treated controls [12,35]. All these results indicate that GnRHa has a significant potential in reducing symptoms, fibroid size and risk of bleeding during surgery. Ulipristal acetate has been reported to attenuate cascade of fibrosis by reducing TGF-β3 mRNA and protein expression with consequent reduction of leiomyoma fibrosis [36]. However, pattern of fibrosis in different uterine leiomyomas and myometrium and changes in the accumulation of fibrosis after hormonal treatment is unclear. A recent study demonstrated that the uteri of post-menopausal women undergo progressive fibrosis with arteriosclerotic changes overtime from the last menstruation [37]. If similar fibrosis occurs in pre-menopausal women of uterine myoma and in perivascular area within myometrium, then we cannot exclude the risk of atherosclerotic risk in these women. This is still an unclear issue for the clinicians to carefully consider when performing surgery for uterine leiomyomas.

Therefore, we aim to investigate the followings for our current study: (1) immunohistochemical analysis of ER and PR expression in fibroid tissue and myometrium derived from GnRHa-treated and -untreated women with SMM, IMM and SSM, (2) analysis of ER and PR expression pattern in intra-tumor/myometrial vascular cells, (3) protein expression of GnRH receptors in and in vitro effect of GnRHa on vascular endothelial cells collected from human umbilical veins (HUVECs), (4) Masson’s trichrome staining with computer-captured image analysis was performed to examine the distribution of fibrosis in myoma/myometrium and in perivascular area derived from GnRHa-treated and -untreated women with different types of fibroids.

## Materials and methods

### Subjects

The subjects in this study were women of reproductive age. This is a prospective non-randomized cohort study. We prospectively collected biopsy samples from women with fibroids and performed retrospective analysis of biopsy samples derived from GnRHa-treated and -untreated women after surgery. Since this is an observational study to examine the effect of GnRHa on expression profiles of ER/PR/fibrosis, we believe that our samples can be considered representative of a larger population. From April 2014 to December 2016, biopsy specimens were collected from a total of 18 women with submucosal myoma (SMM), 16 women with intramural myoma (IMM) and 17 women with subserosal myoma (SSM) who underwent surgery during this period. The criteria for the classification of SMM, IMM and SSM were based on the classification of the European Society of Hysteroscopy [31,38]. All these women were admitted to our hospital with the complaint of abnormal genital bleeding, hypermenorrhoea or anemia with or without associated complaint of dysmenorrhea or pelvic pain. All participants were prospectively recruited based on complains of patients. The uterine fibroids in all these women were diagnosed by ultrasonograghapy and magnetic resonance image before operation. The diagnosis of all fibroids was confirmed by surgery. Six women with SMM, 8 women with IMM and 6 women with SSM were treated with GnRH agonist (leuprolide acetate) for a variable period of 3-6months before operation. Groups of women without GnRHa treatment did not receive oral contraceptives or any other hormonal therapy within 6 months prior to surgery.

The phases of the menstrual cycle in women without hormonal therapy was determined by histological dating of eutopic endometria samples taken simultaneously with removal of myoma nodule. All biopsy specimens were collected in accordance with the guidelines of the Declaration of Helsinki and were approved by the Institutional Review Board of the Research Ethical Committee of Saisaikei Nagasaki Hospital, Nagasaki and Kyoto Prefectural University of Medicine, Kyoto (IRB No. 16005). All patients/samples were recruited/collected in Saiseikai Nagasaki Hospital, Nagasaki and research work was performed in Saiseikai Nagasaki Hospital, Nagasaki/Kyoto Prefectural University of Medicine, Kyoto. All data analysis were performed by an independent investigator (HO) without any prior knowledge of the medical records. All patients in this study received medical treatment during the period of April 2014 and December 2016. All medical records were retrospectively assessed in Saiseikai Nagaskai Hospital during the period of April 2017 and August 2018. A written informed consent was obtained from all women.

### Biopsy specimens

Nine women with SMM and 6 women each with IMM and SSM underwent hysterectomy. Therefore, we could collect myometrial samples only from these women. Trans-cervical hysteroscopic resection (TCR), laparoscopic myomectomy (LM) or laparoscopy-assisted myomectomy (LAM) were performed in the remaining women. Therefore, instead of studying myometrial samples, we could study only pathologic lesions and endometria derived from these women. Biopsy specimens from the myoma nodule, autologous myometria or endometria were collected from all these women during operation. Biopsy specimens obtained after surgery were analyzed for the histological diagnosis of each fibroid. All collected biopsy specimens were prepared for formalin-fixed paraffin-embedded tissue blocks for subsequent histopathological and immunohistochemical study and also for Masson’s trichrome staining with computer-captured image analysis. In case of multiple myoma, biopsy specimen was collected from the largest myoma and analyzed.

### Antibodies used

We performed immunohistochemical studies to investigate immunoreaction of target antigens in the serial section of biopsies using the following antibodies: ERα (estrogen receptor-α, 1:50, NCL-L-ER-6F11, mouse monoclonal, Novocastra Laboratories Ltd, Newcastle, UK); PR (progesterone receptor, 1:40, NCL-L-PGR-1A6, mouse monoclonal, Novocastra Laboratories Ltd, Newcastle, UK); anti-human von Willebrand factor (VWF) antibody (clone F8/86, code M0616; Dako, Denmark, 1:50 dilution), a mouse monoclonal antibody, to investigate immunoreaction to HUVECs. GnRHR (AT2.G7:sc-57176, Santa Cruz Biotechnology, Inc. CA), a mouse monoclonal antibody against both type I and type II receptor (1:25 dilution), was used to immunolocalize GnRHR expression in HUVECs. Non-immune mouse immunoglobulin (Ig) G1 antibody (1:50 dilution, Dako, Denmark) was used as a negative control.

### Immunohistochemistry

The details of immunohistochemical staining procedures are described elsewhere [39,40]. Briefly, five-micrometer thick paraffin-embedded tissues were deparaffinized in xylene and rehydrated in phosphate-buffered saline. After immersion in 0.3% H_2_0_2_/methanol to block endogenous peroxidase activity, sections were pre-incubated with 10% normal goat serum to prevent nonspecific binding and then incubated overnight at 4°C with respective antibody. The slides were subsequently incubated with biotinylated second antibody for 10 minutes, followed by incubation with avidin-peroxidase for 10 minutes and visualized with diaminobenzidine. Finally, the tissue sections were counterstained with Mayer’s hematoxylene, dehydrated with serial alcohols, cleared in xylene, and mounted. We had at least three slides per biopsy for immunohistochemical analysis. The expressions of ER/PR/VWF/GnRHR in fibroid tissue/myometria and in HUVECs were examined. The immunoreaction of ER/PR was expressed as number of ER/PR immunostained cells per high power field (HPF) (x200) and as a mean of five different fields.

### Isolation of HUVECs

Human umbilical vein endothelial cells (HUVECs) were isolated in primary culture and were obtained from female infant umbilical cords of women who underwent normal delivery without complications. A sterile technique was utilized in all manipulations of the cord. HUVECs were separated by collagenase digestion (10mL of 0.2% collagenase) of the interior of the umbilical vein. The separated endothelial cells were collected in 50mL conical centrifuge tube containing 10mL of DMEM medium with 10% fetal bovine serum (FBS). The cells were sedimented at 250g for 10min, washed twice with 10mL of DMEM + 10% FBS and finally yields a range of 0.5–1.5x10^6^ cells. An aliquot of 0.5x10^4^ cells was used for our experiments. The detail procedure of HUVECs isolation and preparation is mentioned in a previous report [41]. HUVECs were cultured at 37°C under 5% CO_2_ in 75cm^2^ tissue culture flasks until the cells reached pre-confluence. Cell purity was confirmed by the appearance of typical cobblestone morphology and reaction to VWF antigen. The cells were re-plated onto 6- or 96-well plates until cells reached pre-confluence. The medium was changed to phenol red depleting DMEM containing 5% charcoal-treated FBS, penicillin and streptomycin, and the cells were pre-cultured for 12h. The cells were then incubated for 24h with medium without and with different doses of GnRHa (10^-9^M to 10^-5^M) (Leuplin: leuprolide acetate, Takeda, Tokyo, Japan). Leuprolide acetate (LA) was dissolved in ethanol to a final concentration of 0.1% per well. We confirmed the validity of these doses of LA in our previous study using EECs/ESCs and SMCs isolated from endometria and myometria of women with uterine myoma [33].

### Cell proliferation assays

5-Bromo-2-deoxyuridine (BrdU) labeling and detection kit measures cell proliferation by quantitating BrdU incorporated into the newly synthesized DNA of replicating cells [42]. The incorporated BrdU can be detected by a quantitative cellular enzyme immunoassay (Biotrak, Amersham Pharmacia Biotech Ltd., UK) using monoclonal antibodies directed against BrdU. It offers a non-radioactive alternative to the [^3^H]-thymidine-based cell proliferation and carries equal sensitivity and specificity [42]. We examined the proliferation of HUVECs in response to variable doses (10^-9^M to 10^-5^M) of GnRH agonist. The detail procedure of BrdU incorporation assay was described previously [43,44]. The results are expressed as percentage of control (GnRHa, 0M).

### Determination of Fibrosis in biopsy samples

In order to determine the presence of fibrosis in the biopsy specimens derived from women with SMM, IMM, SSM, and surrounding myometrium, we performed Masson’s trichrome (MT) staining with aniline blue. All reagents were purchased from the Muto Chemical Co. Tokyo, Japan and staining procedures were followed according to the instruction of the manual as supplied by the Muto Chemical Co. Fibrosis in the respective biopsy samples was identified by dense or filamentous (fiber-like) blue staining (aniline blue) instead of green staining (methyl green) as described previously [45]. Distribution of staining in the biopsy samples derived from respective myoma nodule and myometrum was analyzed. A minimum of five different fields in each MT-stained slide were examined by an independent observer (HO).

### Computer-captured image analysis of fibrosis

The details of computer- captured image analysis of MT-stained fibrosis from each sample is described elsewhere [46,47]. Briefly, for computer-based image analysis, Fiji software was used (ImageJ 2.0.0-rc-61/1.5n, http://fiji.sc). First of all, background correction was done to reduce any color/luminance variations in image. Tissue area (region of interest, ROI) from each stained section was extracted based on luminance information by auto thresholding technique. Automatically calculated threshold was utilized for overcoming non-uniform staining conditions [48]. Dense or filamentous blue-stained area was extracted based on color information. Finally, amount of fibrosis in each tissue specimen was quantified by the following formula: Fibrosis (%) = actual fibrosis area x 100 / ROI area.

### Statistical analysis

All results are expressed as mean ± SD or mean ± SEM, median and inter-quartile range (IQR). The clinical characteristics of the subjects were compared with one-way analysis of variance. The differences in the ER/PR expression and fibrosis in respective biopsy samples between groups and among groups were analyzed by the non-parametric Mann-Whitney *U*-test and Kruskal-Wallis test, respectively. This is an observational study and not a randomized clinical trial. Our main purpose was to examine the effect of GnRHa on the expression profiles of ER/PR/fibrosis in women with uterine myomas comparing to GnRHa-untreated women. Therefore, we did not perform sample size calculation based on pre-specified power in this study. Instead we performed post-hoc power calculation regarding the estimated effect size as the true effect size for Mann-Whitney test in three groups of fibroids. The distribution of ER/PR/fibrosis according to groups was expressed using the box and whisker plots with the medians and IQR. A value of p<0.05 was considered statistically significant. Data analysis was conducted using SAS software version 9.4 (SAS Institute Inc. Cary, NC, USA).

## Results

The women with SSM were significantly younger than those of SMM (36.3 ± 5.2 versus 40.8 ± 8.1 years, p = 0.02). There was no difference in the mean ages between women with SMM and IMM. The mean sizes of SMM were significantly smaller than those of either IMM or SSM (p<0.0001 for each). The distribution of women with or without GnRHa therapy, phases of menstrual cycle and types of surgery in women with SMM, IMM and SSM are summarized in Table 1.

**Table 1:** Clinical profiles of women with different uterine leiomyomas.

	Submucosal (n = 18)	Intramural (n = 16)	Subserosal (n = 17)
Age (yrs.) (mean ± SD)	40.8 ± 8.1	39.1 ± 7.1	36.3 ± 5.2*
Range in age (yrs.)	17–51	28–52	29–44
Size (cm) (mean ± SD)	3.1 ± 0.9**	6.8 ± 2.8	7.0 ± 3.2
Range in size (cm)	1.8–4.5	3.0–12	2.0–14
Solitary/multiple (n)	18/0	14/2	16/1
Menstrual cycle: P/S/M/A (n)	3/8/1/6	2/6/0/8	3/8/0/6
GnRHa therapy: No/yes (n)	12/6	8/8	11/6
Coexistent diseases: None/endo/adeno/dermoid (n)	10/7/1/0	12/3/0/1	11/5/1/0
Surgery performed: TCR/LM/LAM/LAVH/TLH (n)	8/1/0/6/3	0/7/3/6/0	0/11/0/6/0

P, proliferative phase, S, secretory phase, M, menstrual phase, A, amenorrhea; TCR, transcervical hysteroscopic resection, LM, laparoscopic myomectomy, LAM, laparoscopy-assisted myomectomy, LAVH, laparoscopy-assisted vaginal hysterectomy, TLH, total laparoscopic hysterectomy; GnRHa, gonadotropin releasing hormone agonist

*p = 0.02 subserosal vs. submucosal myoma

**p<0.0001, submucosal vs. intramural and subserosal myoma.

### ER and PR expression in myoma and myometrium

**Fig 1:**
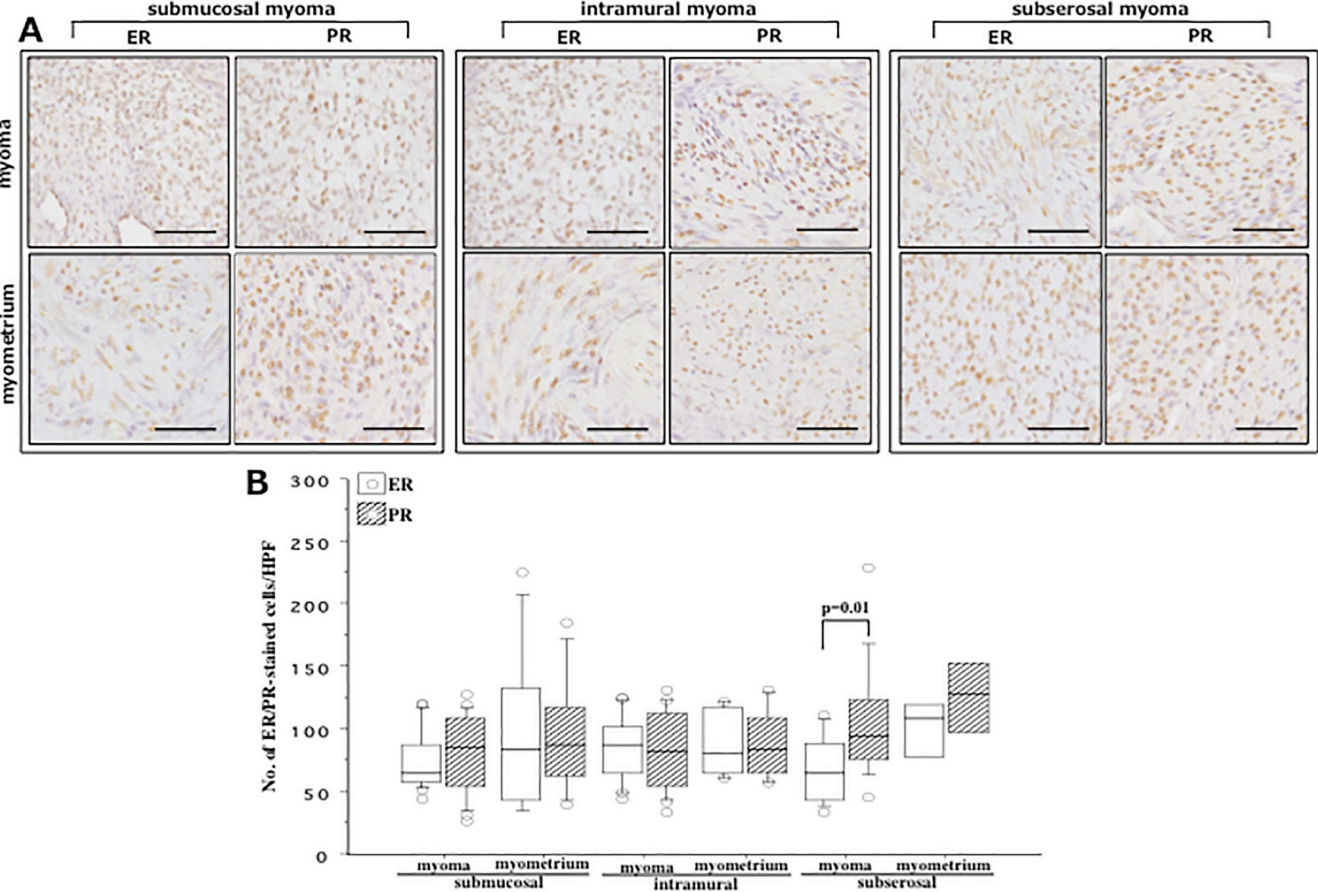
**(A)** Immunohistochemical staining of estrogen receptor (ER) and progesterone receptor (PR) in smooth muscle cells (SMCs) detected in biopsy samples derived from myoma (upper row) and adjacent myometria (lower row) of women with submucosal myoma (left panel), intramural myoma (middle panel) and subserosal myoma (right panel). **(B)** Shows number of estrogen receptor (ER, white box)- and progesterone receptor (PR, hatched box)-immunostained cells in myoma and myometria derived from these three groups of women. Scale bar = 50μm and 100μm for slides of (**A**). Comparing to ER, PR expression was significantly higher in fibroid tissue derived from women with subserosal myoma (p = 0.01). The boxes represent the interquartile ranges and horizontal lines in the boxes represent median values. HPF, high power field (x200).

We analyzed the result of ER and PR expression in respective myoma nodule and myometrium derived from SMM, IMM and SSM regardless of GnRHa treatment (Fig 1A). We found that in women with SSM, PR expression was significantly higher than ER (p = 0.01) without showing any difference between ER and PR in myometrium (Fig 1B). There was no difference between ER and PR expression in either myoma or myometrium derived from women with SMM and IMM (Fig 1B). Kruskal-Wallis test indicated no difference in the expression of ER and PR in either myoma or myometrium among these three groups of women.

### ER and PR expression in vessels within myoma and myometrium

**Fig 2:**
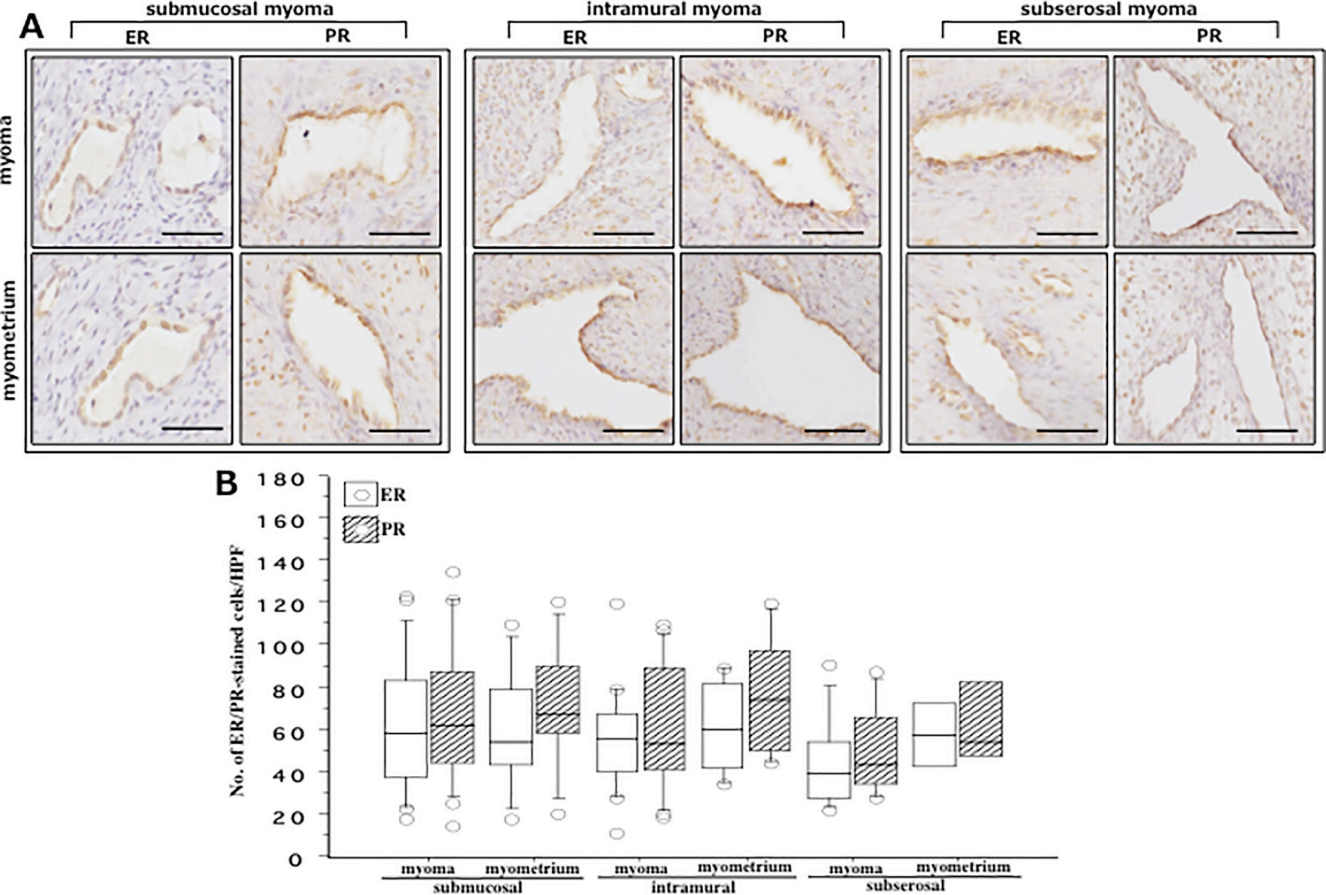
**(A)** Immunohistochemical staining of estrogen receptor (ER) and progesterone receptor (PR) in vascular endothelial cells (VECs) detected in biopsy samples derived from myoma (upper row) and adjacent myometria (lower row) of women with submucosal myoma (left panel), intramural myoma (middle panel) and subserosal myoma (right panel). Scale bar = 50μm for each slide. **(B)** Shows number of estrogen receptor (ER, white box)- and progesterone receptor (PR, hatched box)-immunostained VECs within myoma and adjacent myometria derived from these three groups of women. There was no significant difference between ER-and PR-stained VECs in either myoma or myometria derived from three types of fibroids. The boxes represent the interquartile ranges and horizontal lines in the boxes represent median values. HPF, high power field (x200).

In an attempt to examine the pattern of ER and PR expression in intra-tumor or intra-myometrial vessels, no remarkable change of ER and PR expression was observed in vascular cells in either between groups (myoma and myometrium) or among groups (SMM, IMM and SSM) (Fig 2).

### Distribution of ER and PR expression in myoma and myometrium derived from GnRHa-treated and -untreated women

**Fig 3:**
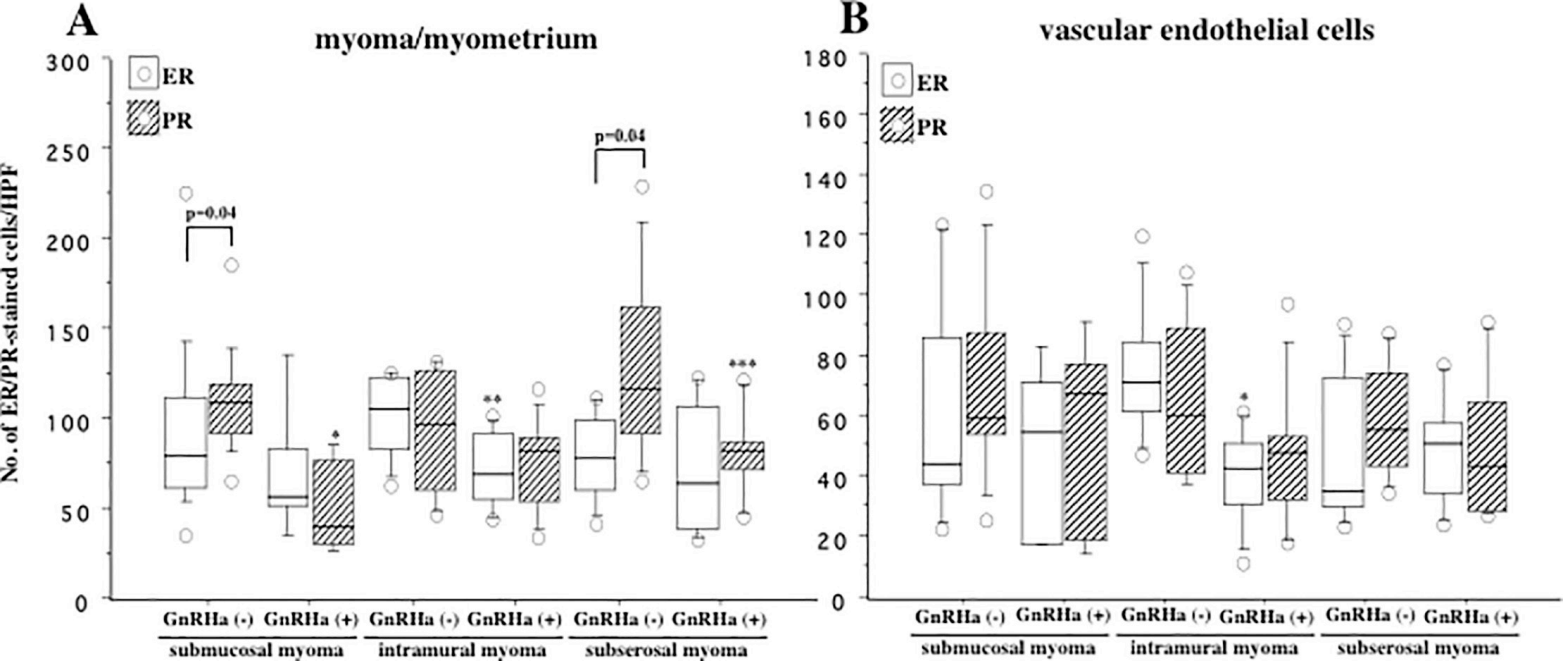
**(A)** Shows the number of estrogen receptor (ER, white box) and progesterone receptor (PR, hatched box)-immunostained cells in the combined myoma and myometria derived from GnRHa-treated (GnRHa+) and GnRHa-untreated (GnRHa-) women with submucosal myoma (SMM), intramural myoma (IMM) and subserosal myoma (SMM). In GnRHa-unteated group, PR content was significantly higher than ER in women with SMM and SSM (p = 0.04 for both). *p = 0.001, GnRHa (+) vs. GnRHa(-) for PR in women with SMM; **p = 0.03, GnRHa (+) vs. GnRHa(-) for ER in women with IMM; ***p = 0.03, GnRHa (+) vs. GnRHa(-) for PR in women with SSM. **(B)** Shows the number of ER and PR-immunostained vascular endothelial cells in the combined myoma and myometria derived from GnRHa-treated (GnRHa+) and GnRHa-untreated (GnRHa-) women with SMM, IMM and SMM. *p = 0.002, GnRHa (+) vs. GnRHa(-) for ER in women with IMM. The boxes represent the interquartile ranges and horizontal lines in the boxes represent median values. HPF, high power field (x200).

We analyzed the results of ER and PR expression in combined myoma and myometrium derived from women with GnRHa-treated and -untreated women with SMM, IMM and SSM. We found that number of PR was significantly higher than ER in myoma/myometria derived from GnRHa-untreated women with SMM (p = 0.04) and SSM (p = 0.04) but not with IMM (Fig 3A). Comparing to untreated groups, GnRHa-treatment significantly decreased PR expression in SMM (p = 0.001) and in SSM (p = 0.03) (Fig 3A). On the other hand, ER expression was significantly decreased in GnRHa-treated women with IMM (p = 0.03) than in GnRHa-untreated women without showing any difference in PR expression between treated and untreated group (Fig 3A).

We further analyzed pattern of ER and PR expression in intra-tumor and intra-myometrial vasculatures in GnRHa-treated and -untreated group of these three types of fibroid. There was no difference between ER and PR content in vascular endothelial cells (VECs) of biopsy samples derived from GnRHa-untreated women with either of SMM, IMM or SSM (Fig 3B). In contrast, we found a significant decrease of ER-immunostained VECs in the GnRHa-treated group with IMM (p = 0.002) than in untreated group without showing any difference in PR expression (Fig 3B). No remarkable change of ER/PR expression was found between GnRHa-treated and -untreated women with SMM and SSM (Fig 3B). Kruskal-Wallis test showed no difference in either ER or PR expression among these three groups.

### Expression of GnRH receptor in HUVECs

**Fig 4:**
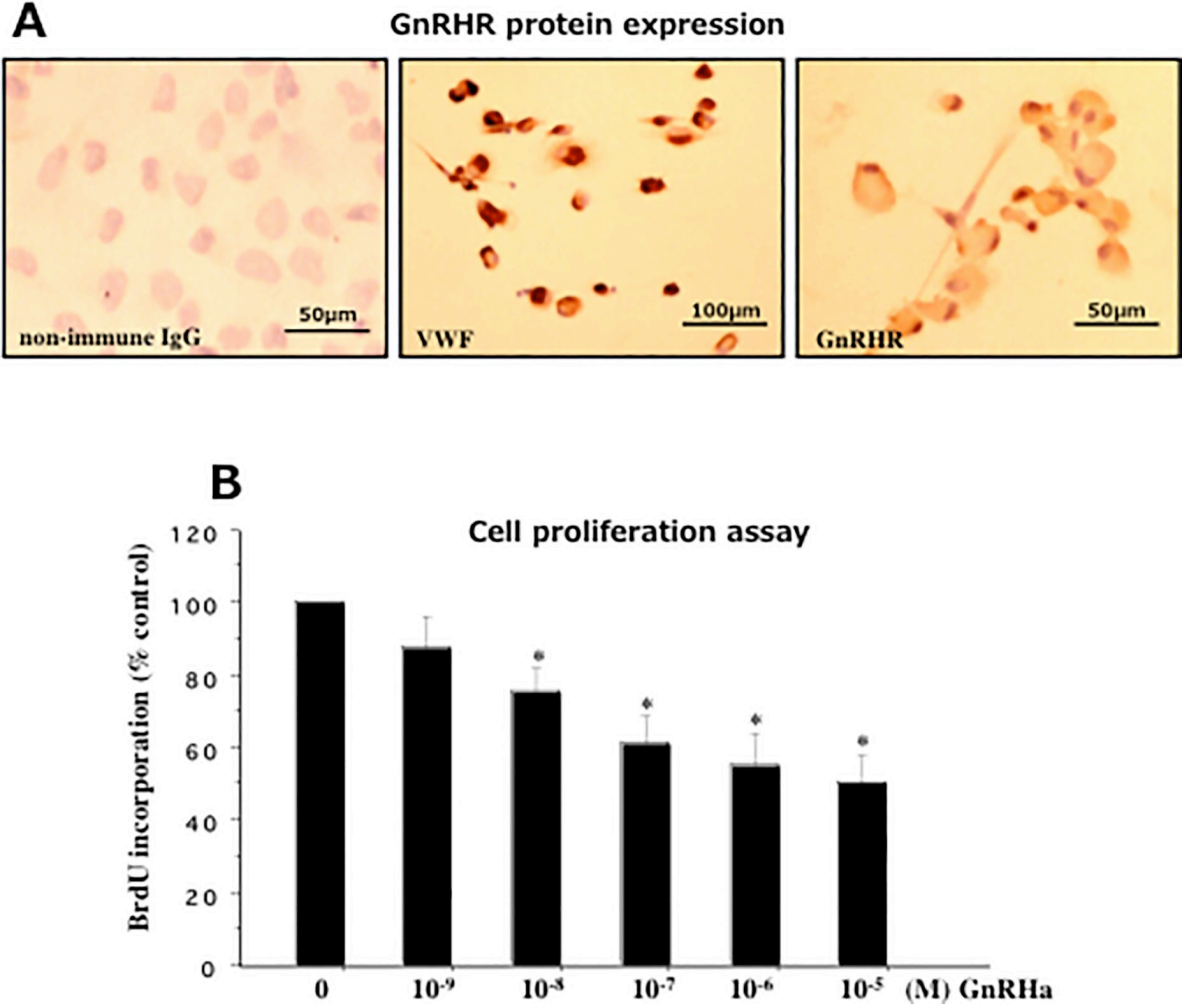
**(A)** Immunohistochemical staining of von Willebrand factor (VWF, middle slide) and gonadotropin-releasing hormone receptor (GnRHR, right slide) in vascular endothelial cells derived from human umbilical vein (HUVECs). The protein expression of GnRHR was detected in VWF-immunostained HUVECs. Non-immune IgG-stained HUVECs is shown as a negative control (left slide). (**B**) Effect of gonadotropin-releasing hormone agonist (GnRHa, leuprolide acetate) on BrdU incorporation into HUVECs. Both GnRHa-treated and non-treated cells were incubated for 48h. Results are expressed as the mean percentages of the untreated cells (± SEM) of triplicate experiments using cells derived from HUVECs. *p<0.05 for each indicated dose (10^-8^M to 10^-5^M) of GnRHa-treated versus -untreated cells.

We found strong immunoreaction of VWF in cultured vascular endothelial cells collected from human umbilical vein (HUVECs) (Fig 4A, middle slide). We also found protein expression of GnRH receptor (GnRHR) in the VWF-immunostained cultured endothelial cells collected from HUVECs (Fig 4A, right slide). GnRHR expression was previously confirmed at the gene level in epithelial cells (EECs), stromal cells (ESCs), smooth muscle cells (SMCs) of endometrium/myometrium and in vascular endothelium [33,49,50].

### Effect of GnRHa on the proliferation of HUVECs

After an initial time-dependent study from day 1 to day 3, we found significant inhibitory response of GnRHa (Leuprolide acetate) on cell proliferation from day 2 to day 3 without any variation between these days. Therefore, our dose-dependent study was performed with an incubation period of 48 hours. We examined direct cell proliferation effect of a variable dose of GnRHa on HUVECs derived from three cases. GnRHa treatment, at a concentration between 10^-8^M and 10^-5^M caused a significant and a dose-dependent inhibition of BrdU incorporation into DNA of HUVECs (p<0.05 for each of 10^-8^M to 10^-5^M of GnRHa-treated cells versus GnRHa-untreated cells) (Fig 4B). Kruskal-Wallis test was used to adjust p-values for multiple comparisons. We found that the maximal anti-proliferative effect was observed at 10^-6^M and 10^-5^M concentration of leuprolide acetate comparing to other doses. We could exclude the cytotoxic effect of GnRHa on HUVECs by trypan blue exclusion test.

### Distribution of fibrosis in myoma and myometrium

**Fig 5:**
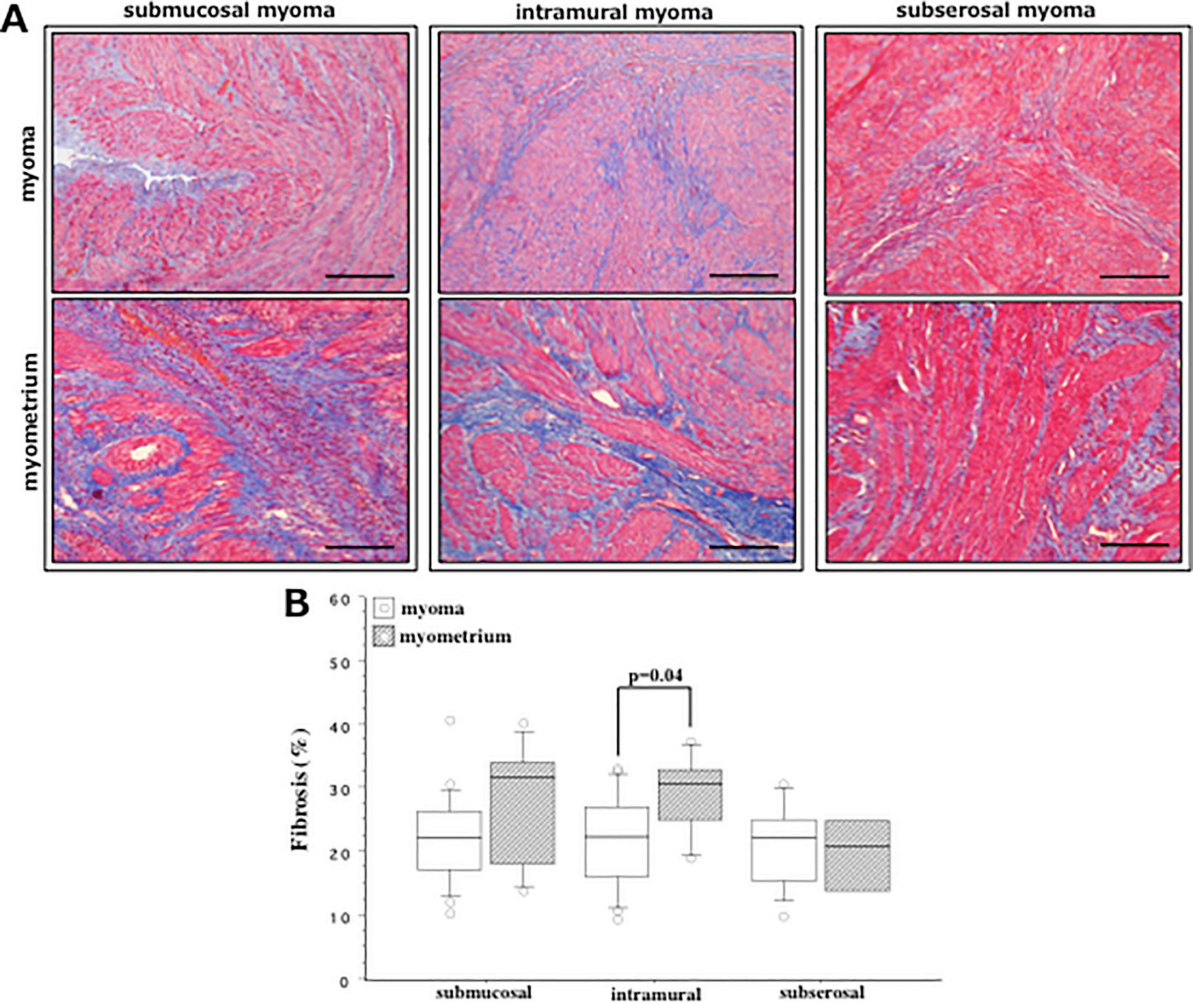
**(A)** Masson’s trichrome (MT)-stained fibrosis in the biopsy samples derived from myoma (upper row) and myometria (lower row) of women with submucosal myoma (SMM, left column), intramural myoma (middle column) and subserosal myoma (SSM, right column) regardless of GnRHa treatment. Scale bar = 50μm for each slide. **(B)** Computer-captured image analysis of fibrosis indicated that comparing to myoma (white box), percentage of fibrosis was significantly higher in myometrium (hatched box) derived from women with IMM (p = 0.04). There was no significant difference in the percentage of fibrosis between myoma and myometria derived from women with SMM and SSM. The boxes represent the interquartile ranges and horizontal lines in the boxes represent median values.

We performed Masson’s trichrome (MT) staining to identify aniline blue-stained filamentous collagen fibers (fibrosis) in myoma and myometria derived from women with SMM, IMM, SSM (Fig 5A). Computer-captured image analysis indicated that percentage of fibrosis was significantly higher in the myometria (median, 30.8%) than in the myoma (median, 22.6%) derived from women with IMM (p = 0.04) (Fig 5B). There was no remarkable difference in the amount of fibrosis between myoma and myometria collected from women with SMM or SSM. The detail percentages of fibrosis in myoma/myometria derived from these three groups of women are shown in S1 Table.

### Distribution of fibrosis in myoma/myometria derived from GnRHa-treated and -untreated women

**Fig 6:**
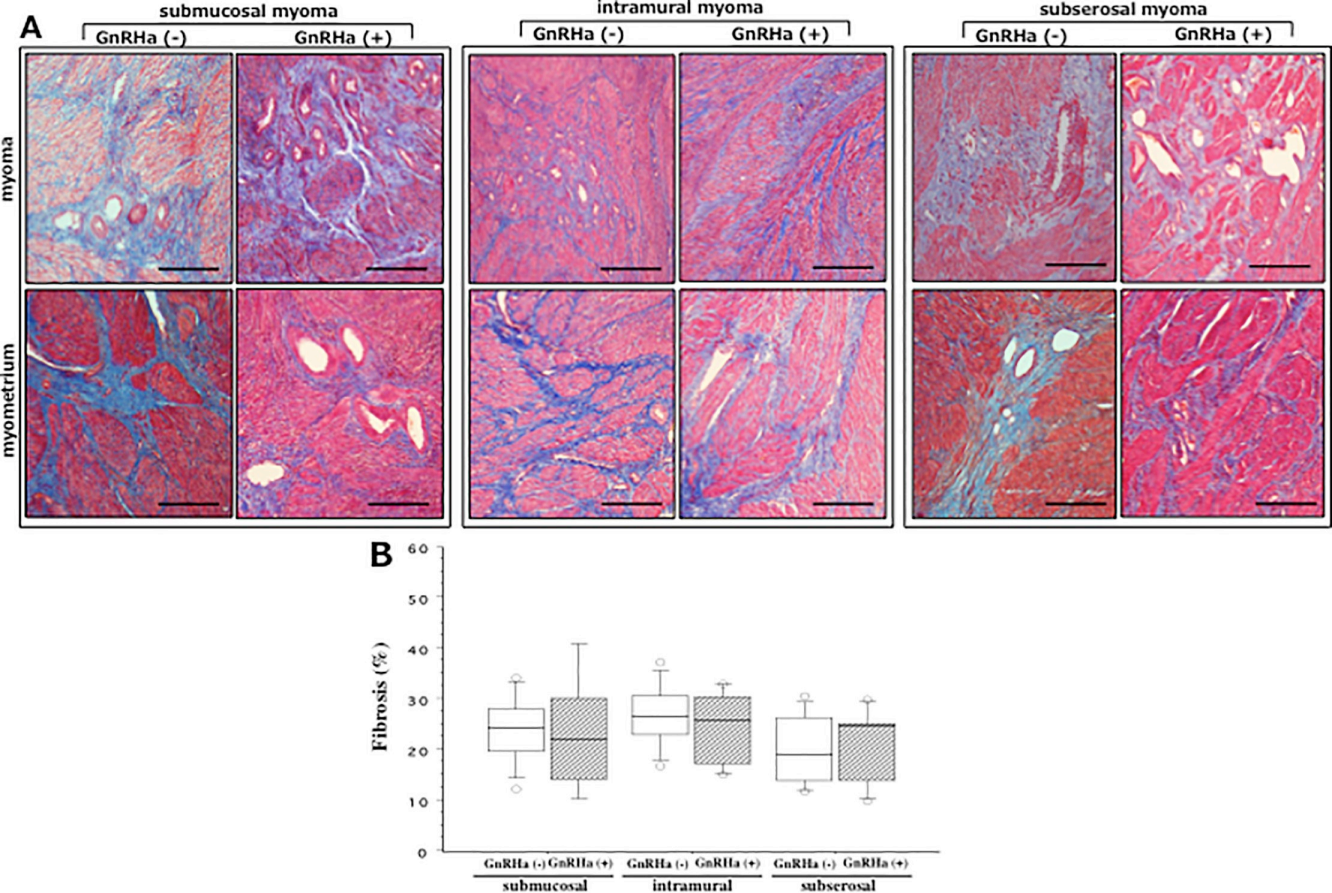
**(A)** Masson’s trichrome (MT)-stained fibrosis in the biopsy samples derived from myoma (upper row) and myometria (lower row) of GnRHa-treated (GnRHa+, hatched box) and GnRHa-untreated (GnRHa-, white box) women with submucosal myoma (SMM, left column), intramural myoma (IMM, middle column) and subserosal myoma (SSM, right column). Scale bar = 50μm for each slide. **(B)** Computer-captured image analysis of fibrosis in combined myoma and myometria indicated that there was no significant difference in the percentage of fibrosis between GnRHa (-) and GnRHa (+) group of women with SMM, IMM and SSM. The boxes represent the interquartile ranges and horizontal lines in the boxes represent median values.

The distribution of fibrosis as measured by Masson’s trichrome-staining in the myoma and myometria derived from GnRHa-treated and -untreated women with SMM, IMM and SSM is shown in Fig 6A. A variable accumulation of moderate to strong aniline blue-stained collagen fibers (fibrosis) was observed in each biopsy specimens derived from these three groups of women. Computer-captured image analysis in order to quantify the total amount of fibrosis showed no significant difference in the percentage of fibrosis in the combined myoma/myometria between GnRHa-treated and -untreated women with SMM, IMM and SSM (Fig 6B). The detail percentages of fibrosis in combined myoma/myometria derived from GnRHa-treated and -untreated women with different types of fibroids are shown in S2 Table.

### Perivascular distribution of fibrosis in myoma and myometrium

**Fig 7:**
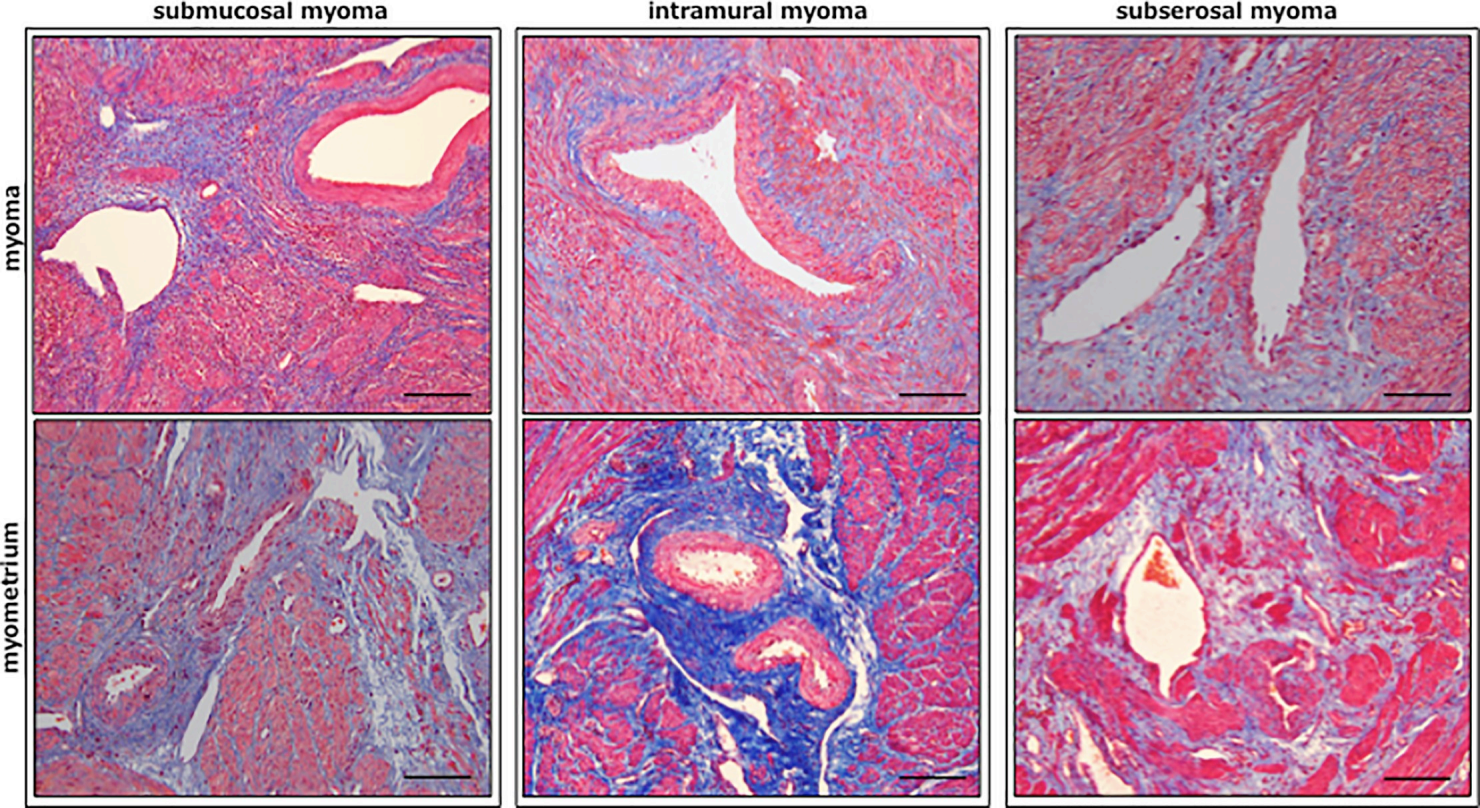
The distribution of Masson’s trichrome (MT)-stained perivascular fibrosis in the biopsy samples derived from myoma (upper row) and myometria (lower row) of women with submucosal myoma (SMM, left column), intramural myoma (IMM, middle column) and subserosal myoma (SSM, right column). A variable distribution of dense fibrosis was observed around the small size and large size vessels in the myoma and myometria derived from women with SMM, IMM and SSM. Scale bar = 50μm for each slide.

The distribution of Masson’s trichrome-stained collagen fibers (fibrosis) was predominant around the small size and large size vessels in the myoma and myometria derived from women with SMM, IMM and SSM (Fig 7). A variable distribution of dense perivascular fibrosis was found in the myoma and myometria derived from these three groups of women.

### Distribution of fibrosis according to age of patients and size of myoma

Masson’s trichrome-stained slides derived from myoma and myometria of all these three groups of women with SMM, IMM and SSM demonstrated an age-dependent increasing distribution of collagen fibers (fibrosis) in their 30^th^, 40^th^ and 50^th^ years comparing to their 10^th^ and 20^th^ years of age (S1 Fig). A similar trend of age-dependent accumulation of fibrosis was also observed in the perivascular area of myoma and myometria in these women (S2 Fig).

**Fig 8:**
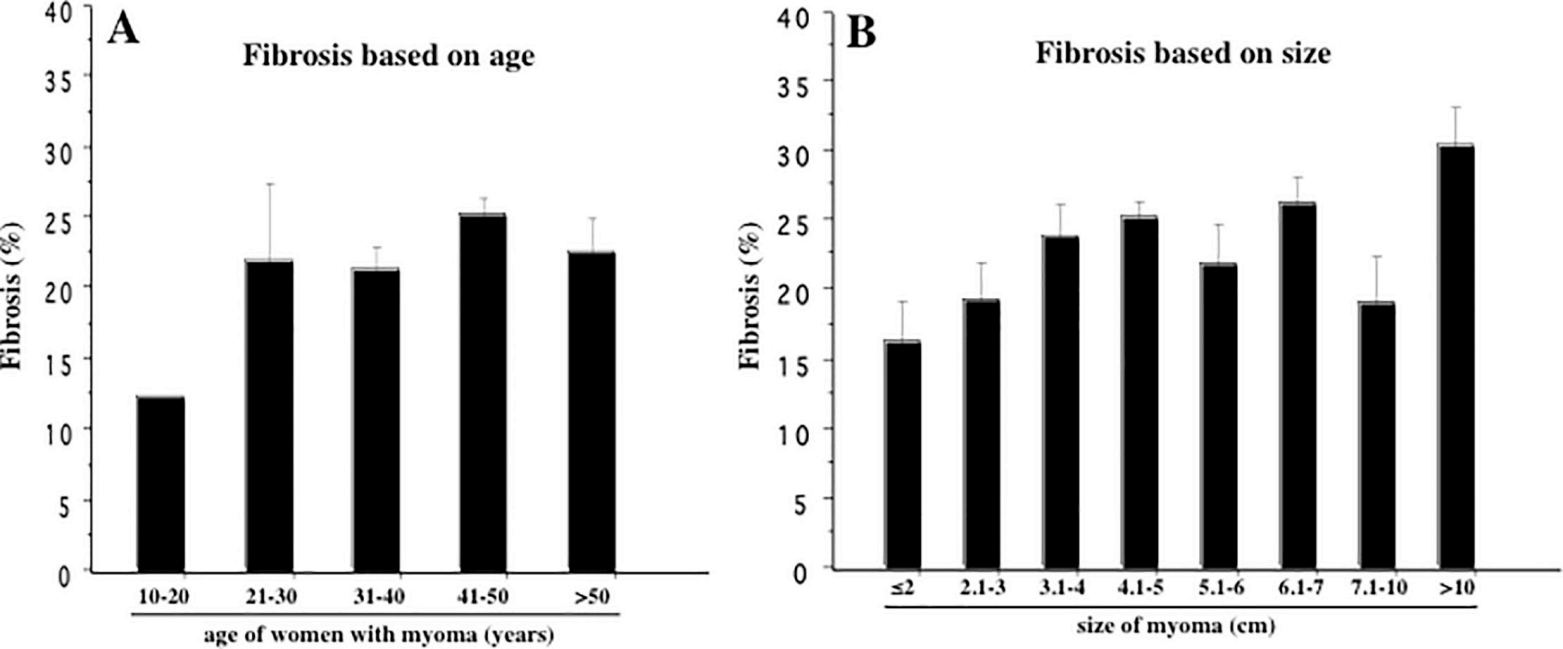
**(A)** Computer-captured image analysis of fibrosis in the biopsy specimens derived from combined myoma and myometria of women with different types of uterine myoma based on age of the patients. The percentage of fibrosis in their 30^th^, 40^th^ and 50^th^ years appears to be higher comparing to women in their 10^th^ and 20^th^ years of age. (**B**). Analysis of fibrosis in similar biopsy specimens demonstrated a size-dependent increase in the percentage of fibrosis in women with combined SMM, IMM and SSM. Kruskal-Wallis test indicated the higher percentage of fibrosis in myoma/myometria with increasing size (4.1-5cm, 6.1–7.0cm and >10cm) comparing to those with other sizes of myoma. The results are expressed as mean ± SEM.

Computer-captured image analysis showed that amount of fibrosis was apparently higher in their 30^th^, 40^th^, and 50^th^ years of age comparing to 10^th^ and 20^th^ years of age (Fig 8A). When we distributed Masson’s trichrome-stained slides derived from myoma and myometria based on the size of fibroids, computer-captured image analysis demonstrated a size-dependent increase in the amount of fibrosis in women with combined SMM, IMM and SSM (Fig 8B). Kruskal-Wallis test indicated the higher percentage of fibrosis in myoma/myometria with increasing size (4.1-5cm, 6.1–7.0cm, and >10cm) comparing to other sizes of fibroids (Fig 8B).

## Discussion

We demonstrated for the first time parallel expression profiles of ER/PR and distribution of fibrosis in myomata, adjacent myometria and vascular endothelial cells (VECs) within these tissues derived from women with different types of fibroids and their response to estrogen suppressing agent. Most of the published reports demonstrated ER/PR expression and individual component of extracellular matrices (ECM) in a specific type of leiomyoma. We came to learn from our study that in GnRHa-untreated women, PR expression was significantly higher than ER in the myoma/myometria derived from women with SMM and SSM. Comparing to GnRHa-untreated women, GnRHa treatment significantly decreased PR expression in biopsy samples collected from SMM and SSM while samples collected from IMM showed a significant decrease of ER expression. We performed post-hoc power calculation regarding the estimated effect size as the true effect size for Mann-Whitney test in three groups of fibroids. We found that the post-hoc power was high for women with SMM (0.908) and relatively low for women with IMM (0.556) and SSM (0.541).

There was no remarkable change of ER/PR content in vasculatures between GnRHa (-) and GnRHa (+) women with SMM and SSM while women with IMM showed a significant decrease of ER expression in the vasculatures after GnRHa treatment. The direct anti-proliferative effect of GnRHa on VECs was confirmed in a separate in vitro study using HUVECs. Expression profile of fibrosis in myoma/myometria collected from with these three groups of fibroids failed to show any remarkable change in the accumulation of fibrosis between GnRHa (-) and GnRHa (+) group. The biopsy specimens of myoma and myometria collected from these three groups of women showed a strong perivascular fibrosis with an apparent increasing tendency with higher age of women and larger size of fibroids.

One outstanding finding of our study is that PR content was predominant in both SMM and SSM and their adjacent myometria. This finding coincides with the results of Nisolle at al., [12] who found statistically significant dominance of ER and PR in leiomyomata and their adjacent myometrium. In our study we did not find any change in the expression of ER in any type of these myomas. The study by Nisolle et al., [12] found a significant decrease of PR content in SMM under GnRHa therapy while ER expression remained unchanged. In our study, we found significant decrease of ER expression in IMM and PR expression in both SMM and SSM after GnRHa treatment. The predominant expression of PR in GnRHa-untreated women with SMM and SSM may suggest that some variants of myoma (SMM/SSM) may be under the influence of progesterone that may play a major role in their growth. This statement might be supported by the findings that women treated with progestogens before hysterectomy showed significantly higher mitotic activity in their fibroids than in women untreated or pretreated with a combined estrogen-progestogen preparation [51]. Another report [52] demonstrated a dramatic increase in leiomyoma size in a menopausal woman taking a high dose of progesterone and a good regression in size when medication was stopped. Ichimura et al., [53] further indicated that both the density and intensity of PR immunostaining in fibroid tissue showed significant positive correlation with leiomyoma size. In addition, the antiprogestogen, RU 486, is as effective as leuprolide acetate in decreasing both uterine volume and blood flow [54]. All these findings including ours further confirmed the role of progesterone in leiomyoma growth despite the traditional view that leiomyomata are only estrogen-dependent. In addition to estrogen, progesterone might be an important factor in the growth of leiomyomata. The enriched PR content may be explained by the fact that leiomyomata may be more sensitive to high concentrations of local estrogen which could provoke a high PR expression.

When we distributed ER- and PR-immunostained SMCs separately in myoma and myometrium derived from women with SMM, IMM and SSM regardless of GnRHa treatment, a predominant expression of PR than ER was observed in the myoma of IMM rather than in adjacent myometrium. There was no difference in the expression profile between ER and PR in either myoma or in myometria collected from women with SMM and SSM. The higher ER content in leiomyomata than in myometrium was reported previously [12,55]. In this study, we detected only ERα in respective tissues using immunohistochemistry. It has been suggested that a correlation exists between ERα and the proliferative activity. Although respective roles of ERα and ERβ remains unclear, Matsuzaki et al., [56] reported that estrogenic effect occur predominantly through ERα and ERβ may play a role in the modulation of estrogenic action. A menstrual phase-dependent and -independent expression of ER and PR in fibroid tissue has been reported [12–15]. After subdividing leiomyomas according to their topographic criteria, as submucosal or subserosal myoma, Marugo et al., [15] observed that ER and PR are significantly more numerous in submucosal than in subserosal myomas, both in the proliferative and secretory phase of the cycle. These findings may agree with the hypothesis that leiomyomas have a different etiology and may explain why some myomas decrease after progestin therapy, while others remain unchanged.

Traditionally medication used to provoke shrinkage of leiomyoma and reduction of blood flow has been based on inducing hypoestrogenism. The only drug that has been shown to be effective is GnRHa (leuprolide acetate). In our previous in vivo and in vitro studies, we demonstrated that in addition to central effect, GnRHa treatment has multiple peripheral effects and was able to significantly decrease tissue inflammation, cell proliferation, angiogenesis, and promotes apoptosis in endometria and myometria derived from women with endometriosis, adenomyosis and uterine leiomyoma [32,33]. In the present study, we found that GnRHa treatment was able to significantly decrease the number of both ER in IMM and PR in SMM and SSM. This indicates that hypoestrogenemia mediated by GnRHa may have a differential functional activity in specific type of myoma through down-regulating either ER or PR. Similar decreased expression of ER/PR and Ki-67 in leiomyoma with consequent decrease of fibroid size has been reported elsewhere [12,35,57]. Our current findings of decreased ER/PR expression in response to leuprolide acetate confirmed the previous findings.

When we analyzed pattern of ovarian steroid receptors in vascular endothelial cells (VECs) within myoma/myometria, no significant difference between ER and PR expression was found in GnRHa-untreated women with different fibroids. Distribution of ER/PR expression profile separately in the myoma and myometria derived from different fibroids and regardless of GnRHa treatment showed no significant difference between ER and PR in either myoma or in myometrium. Comparing to GnRHa-untreated group, the only significant difference was observed for vascular ER expression in women with IMM after GnRHa treatment. This might to be due to our staining performance with ERα and not with ERβ. Most of the previous studies reported ERβ expression in pregnant and non-pregnant uterine vascular endothelial cells instead of ERα expression [58,59]. However, using ovine uterine artery endothelial cells (UAECs) in vivo and in vitro, Liao et al., [60] demonstrated both ERα and ERβ expression in UAECs. This differential expression of ERα and ERβ in VECs may indicate a direct target of estrogenic action in VECs mediated by either ERα or ERβ. Another interesting study informed us that human endothelial progenitor cells (EPCs) express ERα mRNA and protein and estrogen stimulates proliferation of EPCs via ERα [61]. We speculate that there might be a shifting of ERα to ERβ content in VECs due to time-dependent differentiation and maturation of EPCs into UAECs.

We previously demonstrated a significant decrease in micro-vessel density as measured by VWF immunostaining in leiomyomata and adjacent myometrium in response to GnRHa treatment [32]. In an attempt to investigate the direct anti-proliferative effect of GnRHa on VECs, we found that similar to EECs/ESCs and myometrial SMCs [33], HUVECs also expressed GnRHR at protein level and in vitro treatment with GnRHa significantly and dose-dependently decreased cell proliferation of HUVECs. The collective findings of current and previous studies may explain the mechanistic basis of reduction in fibroid size, and reduced risk of bleeding during surgery after GnRHa treatment. A controversial debate still persists whether we should use GnRHa before myoma surgery or not. Although not common, GnRHa is still clinically used with the preference of patients and in order to alleviate manifestations, minimize risk of intra-operative bleeding and to reduce operative time.

One of the major disadvantages of using GnRHa in treating uterine leiomyoma is ineffective reduction in size and recurrence of the disease once treatment is stopped. In fact, most leiomyomata return to pretreatment size within 4months following cessation of therapy. This can be explained by withdrawal of HPO-axis down-regulation and stiffness of the uterine wall resulting from dense accumulation of ECM components within myometria and in perivascular area. Fibroids contain substantial amounts of altered and disordered collagens that contribute to their bulk. Rigidity of the uterus is an important factor that may also explain the poor responsiveness to hormonal medication [46]. A variable amount of tissue inflammatory reaction in the myometrium with consequent accumulation of collagen fibers, collagen matrix and fibrous elements may time-dependently cause fibrosis and stiffness of the myometrial tissue [9,46,47]. With this background in mind, we were curious to know the distribution of fibrosis in the leiomyomata, adjacent myometrium and their perivascular area derived from GnRHa-treated and -untreated women with different uterine leiomyomas.

We demonstrated in our current study that unlike SMM and SSM, the amount of fibrosis was significantly higher in the myometria than in leiomyomata collected from women with IMM. No significant difference was observed in the percentage of fibrosis in combined myoma/myometria collected from GnRHa-treated and -untreated women with these three types of fibroids. We also found a strong perivascular fibrosis within myoma and myometria in any type of fibroids. The distribution of fibrosis in myoma/myometria and in their perivascular area showed an increasing pattern with higher age of patients and larger size of fibroids. Several lines of evidence supported fibrotic character of uterine leiomyomas by elevated levels of ECM components and myofibroblasts in these tumors [9,62]. Estrogen-and progesterone-mediated up-regulation of ECM proteins such as collagen 1, fibronectin and versican has been demonstrated in fibroids [62]. Using bio-culture system, the authors examined the effect of leuprolide acetate and cetrorelix acetate on these ECM proteins and found that both these hormonal compounds significantly decreased amount of ECM proteins [62]. These results did not coincide with our findings showing that fibrosis remains unchanged in intact tissue after GnRHa treatment. We can explain this discrepancy as follows: (1) as a specific marker of smooth muscle cells, while Desmin stains other areas of myometrium, it skips the area occupied by fibrosis [46]. These findings indicate that rigidity of the uterus resulting from fibrosis may be another potential factor to clarify the hormonal resistance to fibrosis. This may ultimately cause non-effective decrease in uterine size after hormonal medication, (2) while most of the other studies examined in vitro effect of hormonal therapy on the individual components of ECM, we investigated the effect of GnRHa on the highly accumulated ECM and collagen fibers with resultant visible fibrosis in biopsy specimens. Recently, ulipristal acetate (GnRHa antagonist) shows promising effect on inhibiting leiomyoma fibrosis via decreasing TGF-β3 and its canonical signaling pathway as well as by limiting cell proliferation and ECM remodeling [36,63,64].

Another interesting finding in our study is the occurrence of perivascular fibrosis around small size and large size vessels in the myoma and myometrium derived from different types of fibroids. This finding may explain a link in the initiation and extension of fibrosis in the myoma/myometrium of women with uterine leiomyomas. A variable amount of tissue inflammatory reaction in fibroids may produce abundant concentration of TGF-β from infiltrating immune cells and induce a cascade of EMT, FMT, SMM and activation of myofibroblasts ultimately leading to fibrosis [20,22,26–28]. A variable amount of TGF-β in the vasculatures of myometrium may induce a similar event of FMT around the vessels. Once newly generated myofibroblasts around the vessels, even in small size, are activated, they induce collagen production, recruit collagen fibers and ultimately cause perivascular fibrosis [65]. A persistent myofbroblast activity in response to tissue injury and repair causes accumulation and contraction of collagenous ECM, a condition called fibrosis. Starting from the area around the vessels, this fibrotic process may time-dependently extend to other parts of the myoma/myometria and finally cause rigid uterus in women with uterine fibroids.

From our findings of perivascular fibrosis in women with different types of fibroid in premenopausal women, we cannot exclude the possibility of sclerotic change in vessels that may increase the risk of thrombotic events as well as hypoxic change of the endometrium. In fact, we found that deposition of collagen fibers in myoma/myometria and in perivascular areas appear to be higher with advancing age of women comparing to younger age groups. Large size fibroid and associated fibrosis can be another predisposing factor. More recently, it has been reported that the uteri of post-menopausal women undergo progressive fibrosis with atherosclerotic changes overtime from the last menstrual period [37]. If similar fibrosis occurs in pre-menopausal women with uterine myoma, then we cannot exclude the risk of atherosclerotic events in these women. Future study is warranted to confirm this association.

There are several limitations in our current study. (1) These findings are limited by the cross-sectional nature of the study and we used only histochemistry and immunohistochemistry. (2) Sample size in each group of GnRHa-treated and -untreated women with fibroid was small. Further investigation is needed with large sample size. (3) Neither specific types of ER (ERα/ERβ) nor PR (PR-A/PR-B) in respective fibroids were analyzed. (4) HUVECs were used in current study instead of uterine arterial endothelial cells in culture system. (5) We examined only the effect of estrogen suppressing agent on E/P receptor expressions and fibrosis. Additional studies on the effect of different progestational compounds are needed to support our current findings.

Finally we conclude that our current findings give us some new information on the biological differences in three different types of uterine leiomyoma and on the biological basis of ineffective reduction of fibroid size after GnRHa treatment. Our current findings re-confirmed higher PR content in fibroids and may suggest a potential role of progesterone in leiomyoma growth. GnRHa therapy may transiently shrink fibroids and reduce the risk of bleeding during surgery by decreasing ER/PR expression and proliferation of vascular endothelial cells. The most recent report indicated that after adjusting different confounding factors, size, number of fibroids, and operative time were directly related to risk of bleeding during myoma surgery [66]. These clinical factors should be considered to select hormonal therapy before surgery in order to minimize intra-operative risk of bleeding.

The occurrence of myometrial/perivascular fibrosis may impair effective reduction of fibroid size and increase the risk of arteriosclerosis. We need careful follow-up of these women with fibroids even after uterus-sparing surgery such as uterine artery embolization [67]. Based on our current findings, we postulate that use of anti-fibrotic agents either alone or in combination with GnRHa may be the potential target in the treatment of fibroids. The introduction of drugs that are specifically anti-fibrotic could be a good solution to control abnormal leiomyoma growth and associated clinical symptoms. In fact, interest has recently focused on anti-fibrotic therapeutic strategies aimed at blocking factors that directly control myofibroblast activation [68]. High collagen content in uterine fibroid was effectively digested by highly purified collagenase C histolyticum, resulting in reduced tissue stiffness [69]. As a natural compound, effect of vitamin D3 on reducing TGF-β3-induced fibrosis could be another attractive approach in the treatment of uterine leiomyoma [70]. Further studies are required to strengthen our current findings and to confirm the effect of these anti-fibrotic agents either alone or in combination with GnRHa on different fibroids.

## Supporting information

S1 Fig Masson’s trichrome (MT)-stained fibrosis in the biopsy samples derived from myoma (upper row) and adjacent myometria (lower row) of women with submucosal myoma, intramural myoma and subserosal myoma based on the age of the patients regardless of type of fibroid and therapy. An increasing accumulation of fibrosis was observed in patients in their 30^th^, 40^th^ and 50^th^ years age comparing to patients in their 10^th^ and 20^th^ years of age. Scale bar = 50μm for each slide.
(TIFF)

S2 Fig The distribution of Masson’s trichrome (MT)-stained perivascular fibrosis in the biopsy samples derived from myoma (upper row) and adjacent myometria (lower row) of patients with submucosal myoma, intramural myoma and subserosal myoma based on the age of the patients regardless of type of fibroid and therapy. An increasing accumulation of dense perivascular fibrosis was observed in patients in their 30^th^, 40^th^ and 50^th^ years of age comparing to patients in their 10^th^ and 20^th^ years of age. Scale bar = 50μm for each slide.
(TIFF)

S1 Table Distribution of fibrosis in myoma and myometrium regardless of therapy. (DOCX)

S2 Table Distribution of fibrosis in combined myoma/myometrium based on therapy. (DOCX)
