# Supplementary figures and images for: Expression profiles of E/P receptors and fibrosis in GnRHa-treated and -untreated women with different uterine leiomyomas
Source: PLoS One. 2020 Nov 13;15(11):e0242246. doi: 10.1371/journal.pone.0242246 (PMC7665806; doi:10.1371/journal.pone.0242246)

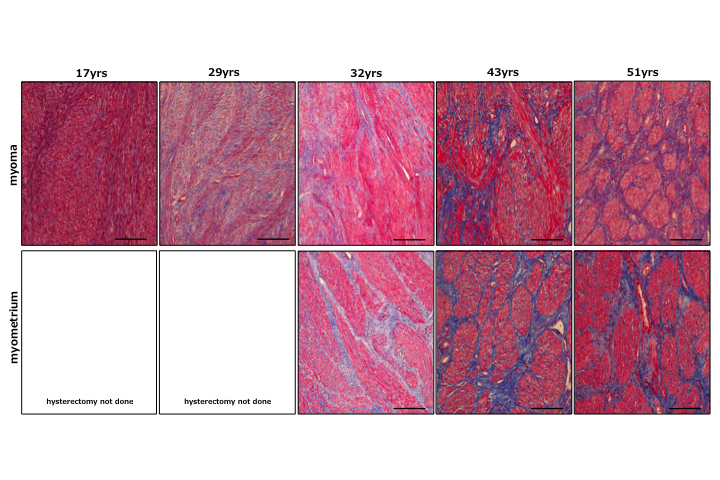

Supplement: S1 Fig — An increasing accumulation of fibrosis was observed in patients in their 30th, 40th and 50th years age comparing to patients in their 10th and 20th years of age. Scale bar = 50μm for each slide. (TIFF) [file pone.0242246.s001.tiff]

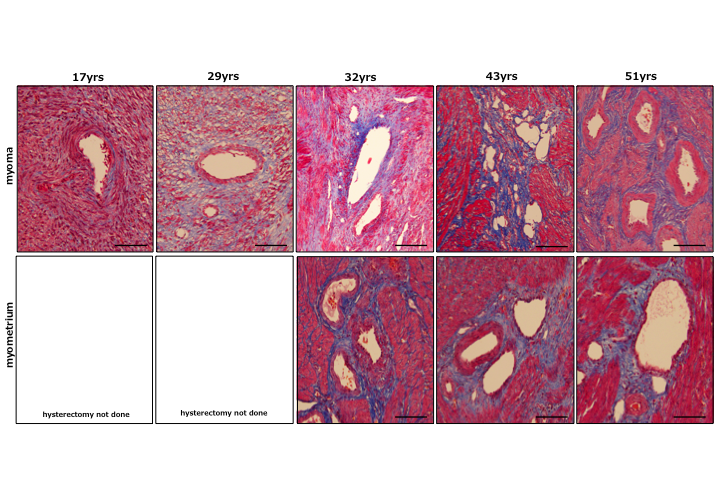

Supplement: S2 Fig — An increasing accumulation of dense perivascular fibrosis was observed in patients in their 30th, 40th and 50th years of age comparing to patients in their 10th and 20th years of age. Scale bar = 50μm for each slide. (TIFF) [file pone.0242246.s002.tiff]
