# Supplementary material for: Expression profiles of E/P receptors and fibrosis in GnRHa-treated and -untreated women with different uterine leiomyomas
Source: PLoS One. 2020 Nov 13;15(11):e0242246. doi: 10.1371/journal.pone.0242246 (PMC7665806; doi:10.1371/journal.pone.0242246)
Supplement: S1 Table — (DOCX) [file pone.0242246.s003.docx]

S1 Table . Distribution of fibrosis in myoma and myometrium regardless of therapy.

| Submucosal Intramural Subserosal  (n=18) (n=16) (n=17) |
| --- |
| Myoma (mean ± SEM) 22.7 ± 7.3 22.0 ± 7.2 20.6 ± 6.1  Median (%) 22.2 22.6 21.1  Range in fibrosis (%) 10.5-40.9 9.7-33.2 10.1-30.8    Myometrium (mean ± SEM) 27.6 ± 9.9 29.4 ± 6.4* 19.2 ± 5.1  Median (%) 31.9 30.8 19.7  Range in fibrosis (%) 14.0-40.3 19.1-37.4 11.8-26.2 |
|  |

The results are expressed as mean ± standard error of mean (SEM) and median.

Intramural myoma, *p=0.04, myometrium versus myoma.
