# Supplementary material for: Expression profiles of E/P receptors and fibrosis in GnRHa-treated and -untreated women with different uterine leiomyomas
Source: PLoS One. 2020 Nov 13;15(11):e0242246. doi: 10.1371/journal.pone.0242246 (PMC7665806; doi:10.1371/journal.pone.0242246)
Supplement: S2 Table — (DOCX) [file pone.0242246.s004.docx]

S2 Table . Distribution of fibrosis in combined myoma/myometrium based on therapy.

| Submucosal Intramural Subserosal  (n=18) (n=16) (n=17) |
| --- |
| GnRHa (-) (mean ± SEM) 23.7 ± 6.9 26.9 ± 6.3 20.1 ± 6.0  Median (%) 23.3 26.6 20.7  Range in fibrosis (%) 12.3-33.5 16.9-37.4 11.8-30.8    GnRHa (+) (mean ± SEM) 22.7 ± 10.5 24.5 ± 7.1 21.4 ± 7.7  Median (%) 24.4 26.0 24.7  Range in fibrosis (%) 10.5-40.9 15.3-33.2 10.1-30.1 |
|  |

The results are expressed as mean ± standard error of mean (SEM) and median.

GnRHa, gonadotropin-releasing hormone agonist.
